# Racial and Ethnic Heterogeneity in Self-Reported Diabetes Prevalence Trends Across Hispanic Subgroups, National Health Interview Survey, 1997–2012

**DOI:** 10.5888/pcd13.150260

**Published:** 2016-01-21

**Authors:** Cassandra Arroyo-Johnson, Krista D. Mincey, Nicole Ackermann, Laurel Milam, Melody S. Goodman, Graham A. Colditz

**Affiliations:** Author Affiliations: Krista D. Mincey, Xavier University, New Orleans, Louisiana; Nicole Ackermann, Laurel Milam, Melody S. Goodman, Graham A. Colditz, Washington University in St Louis, St Louis, Missouri.

## Abstract

**Introduction:**

We examined racial/ethnic heterogeneity in self-reported diabetes prevalence over 15 years.

**Methods:**

We used National Health Interview Survey data for 1997 through 2012 on 452,845 adults aged 18 years or older. Annual self-reported diabetes prevalence was estimated by race/ethnicity and education. We tested for trends over time by education and race/ethnicity. We also analyzed racial/ethnic and education trends in average annual prevalence.

**Results:**

During the 15 years studied, diabetes prevalence differed significantly by race/ethnicity (*P* < .001) and by Hispanic subgroup (*P* < .001). Among participants with less than a high school education, the 5-year trend in diabetes prevalence was highest among Cubans and Cuban Americans (β_5YR_ = 4.8, *P* = .002), Puerto Ricans (β_5YR_ = 2.2, *P* = .06), non-Hispanic blacks (β_5YR_ = 2.2, *P* < .001), and non-Hispanic whites (β_5YR_ = 2.1, *P* < .001). Among participants with more than a high school education, non-Hispanic blacks had the highest average annual prevalence (5.5%) and Puerto Ricans had the highest 5-year trend in annual diabetes prevalence (β_5YR_ = 2.6, *P* = .001).

**Conclusions:**

In this representative sample of US adults, results show ethnic variations in diabetes prevalence. The prevalence of diabetes is higher among Hispanics than among non-Hispanic whites, unevenly distributed across Hispanic subgroups, and more pronounced over time and by education. Findings support disaggregation of data for racial/ethnic populations in the United States to monitor trends in diabetes disparities and the use of targeted, culturally appropriate interventions to prevent diabetes.

## Introduction

Diabetes continues to be a public health problem in the United States (1). The Centers for Disease Control and Prevention (CDC) reports more than 25 million (11.3%) Americans aged 20 years or older have diabetes and 11.8% of Hispanics have diagnosed diabetes (1). Type 2 diabetes accounts for 90% to 95% of all diagnosed cases and currently ranks seventh among the leading causes of death in the United States (1). Disparities in diabetes rates for non-Hispanic whites, non-Hispanic blacks, and Hispanics persist. Specifically, recent estimates show that the risk of diagnosed diabetes is 66% higher for Hispanics than for non-Hispanics (1).

The Hispanic population is the fastest growing group in the United States and consists of people from approximately 25 countries (2). Recent population projections suggest that Hispanics will account for at least 67% of the total population growth during 2015–2060 (3). The 5-year projected percentage growth of Hispanics from 2015 to 2020 is 12.39% and is projected to be 7.74% for 2055–2060 (3). Because of the projected growth of the Hispanic population, the United States can expect increases in diabetes incidence, prevalence, and diabetes-related medical expenditures.

Numerous studies report that Hispanics in the United States have health outcomes that are better than or equivalent to the health outcomes of non-Hispanic whites, despite having lower income and less education (4–12). This phenomenon is referred to as the Hispanic Paradox. Although many ethnicities exist within the Hispanic population, Hispanics in the United States are often placed into pan-ethnic groups for health research. Studies show ethnic variations in the mortality, health outcomes, behaviors, risk factors, demographics, and social determinants of Hispanics (2,4,13–16). When grouped into a pan-ethnic category of “Hispanic” or “Latino,” these variations are masked and the factors affecting the variations across the different subgroups, such as socioeconomic status, are overlooked (2,4,13,15,17–19).

Socioeconomic status (SES) is 1 factor used to explain ethnic variations among Hispanics, but research on the relationship between SES and health outcomes has mixed results (19,20). Schneiderman et al reported finding an association between education and diabetes prevalence — the prevalence of diabetes decreased among women as education and household income increased — but diabetes prevalence for men showed no consistent trend (20). Overall, Puerto Ricans with less than a high school diploma, other Hispanic groups with a high school diploma or general equivalency diploma (GED), and Mexicans with some college education had increased odds of having diabetes (19). However, another study of a diverse group of Hispanics reported that the lower the income or education, the higher the rates of health conditions such as diabetes and obesity (21). These findings demonstrate how various SES factors affect health across the different Hispanic groups. By understanding which subgroups are affected by modifiable SES factors such as education, tailored interventions can be developed to target these groups, which may help decrease the disparities seen within the Hispanic population.

Rather than pooling several years of data to increase sample size, we chose to examine heterogeneity in health outcomes over time for Hispanic subgroups (5,13,15,19–22). Our goals for this study were to 1) describe the heterogeneity within the Hispanic population for self-reported diabetes over time; 2) estimate time trends in the prevalence of self-reported diabetes compared with that of non-Hispanic whites and non-Hispanic blacks; and 3) identify disparities in the prevalence of diabetes over time among Hispanic subgroups, non-Hispanic whites, and non-Hispanic blacks.

## Methods

### Study population

For the purpose of this study, we used National Health Interview Survey (NHIS) data from 1997 to 2012. NHIS consists of annual face-to-face household interviews during which data are collected on health and other characteristics from each member of the household. During the interview, adults answer questions on their demographic characteristics, Hispanic ethnicity, Hispanic subgroup, race, and health-related outcomes, behaviors, and exposures. Methods for NHIS data collection are described in detail elsewhere (23,24).

Currently, NHIS is the largest publicly available national data set with an administered item to distinguish Hispanic subgroups. Before 1999, respondents were asked “Do any of these groups represent (your/name’s) national origin or ancestry?” with the following options: Puerto Rican, Cuban, Cuban American, Mexican/Mexicano, Mexican American, Chicano, Hispanic, other Latin American, and other Spanish or Hispanic. In 1999, the question was revised to first ask “{Do/Does (you/name)} consider {yourself/himself/herself} to be Hispanic or Latino?” and, if the answer was yes, the respondent was asked to indicate their Hispanic origin or ancestry by checking one of the options listed on a form. The options were Puerto Rican, Cuban/Cuban American, Dominican (Republic), Mexican, Mexican American, Central or South American, other Latin American, other Hispanic/Latino, refused, and don’t know. Because of concerns about annual sample size for respondents identifying as Dominican and Central or South American and for consistency in the administered question over the 15-year period, we used the 3 largest subgroups (Mexicans/Mexican Americans, Puerto Ricans, and Cubans/Cuban Americans) and excluded the remaining Hispanic subgroups from the study (23). The study population consists of NHIS respondents aged 18 or older who self-identify as Puerto Rican, Mexican or Mexican American, Cuban or Cuban American, non-Hispanic black, or non-Hispanic white. Pregnant women were excluded from the study.

The outcome for this study is self-reported diabetes. The diabetes question is “[If female, other than during pregnancy] Have you ever been told by a doctor or health professional that you have diabetes or sugar diabetes,” and the response options are yes, no, borderline, refused, not ascertained, and don’t know (19). Participants answering borderline, refused, not ascertained, and don’t know were excluded from the analysis to reduce the potential for misclassification of participants with prediabetes. Sex was also self-reported as male or female. Level of education was categorized as less than a high school diploma, high school diploma or GED, and more than a high school education. Health insurance coverage was dichotomized into the 2 categories: yes (any type of health insurance) or no (no insurance at all).

### Statistical analysis

We calculated descriptive statistics of participant characteristics. For categorical variables, χ^2^ tests of association between participant characteristics and race/ethnicity and Hispanic subgroup were used and analysis of variance was used for continuous variables. Annual prevalence rates for diabetes were calculated for all racial/ethnic groups for 1997 through 2012 using the annual weights developed according to the NHIS weighting scheme, which include poststratification adjustments using Census population control totals. Estimates generated from different years within the same sampling design period are dependent because of the complex sampling scheme. For our analysis, there are 2 design periods: 1997–2004 and 2005–present. Following recommendations from the National Center for Health Statistics (NCHS), we divided final sample weights in each design period by the number of years used for that design period to correct for this dependence (23,24). Annual prevalence of diabetes by race/ethnicity and Hispanic subgroup stratified by education was calculated. Average annual diabetes prevalence was estimated and analysis of variance was used to test the relationship between race/ethnicity and Hispanic subgroup and diabetes prevalence first, and was then stratified by education. Multiple comparisons were conducted to determine significant differences in the average annual diabetes prevalence for race/ethnicity and Hispanic subgroups, using Bonferroni correction of the *P* values. We also tested for an association between level of education and diabetes prevalence stratified by race/ethnicity and conducted multiple comparisons by level of education. To address the hypothesis that changes in prevalence over time vary by race/ethnicity and Hispanic subgroup, we tested for a trend over time in annual diabetes prevalence stratified by race/ethnicity and Hispanic subgroup by using linear regression with year as an ordinal predictor variable. Time trend model intercepts (β_0_) are reported as an estimate of baseline diabetes prevalence in 1997. The estimated changes in diabetes prevalence over 5 years (β_5YR_) are reported, with *P* values, and interpreted as the change in diabetes prevalence for every 5-year increase in time. We used SAS software version 9.4 (SAS Institute, Inc). The Washington University School of Medicine Human Research Protection Office determined that our use of the NHIS data was not subject to institutional review board oversight.

## Results

We present demographic characteristics of the participants for years 1997–2012 combined (Table 1). For that time period, data on 427,975 Hispanic, non-Hispanic white, and non-Hispanic black participants were selected for the analysis. Of those whose data were selected, 14.2% identified as Hispanic, 69.8% as non-Hispanic white, and 16.0% as non-Hispanic black. Among Hispanics, 79.2% self-identified as Mexican/Mexican American, 13.5% as Puerto Rican, 7.3% as Cuban/Cuban American. Mean age in years differed significantly by race/ethnicity (*P* < .001) and Hispanic subgroup (*P* < .001). Although the mean age in years for all Hispanics (mean, 39.6; standard deviation [SD], 0.13) was lower than the mean age for non-Hispanic blacks (mean, 42.8; SD, 0.13) and non-Hispanic whites (mean, 47.5; SD, 0.04), Cubans/Cuban Americans had the highest mean age (mean, 49.2; SD, 0.36) of all racial/ethnic groups. In the racial and ethnic subgroups, there were more women than men except for Mexicans/Mexican Americans and Cubans/Cuban Americans.

**Table 1:** Characteristics of Participants: Non-Hispanics Whites, Non-Hispanic Blacks, and Subgroups of Hispanics, Study of Heterogeneity in Self-Reported Diabetes Prevalence Trends Across 3 Hispanic Subgroups, NHIS 1997–2012^a^

Characteristic	Total	Non-Hispanics (n = 367,292)		Hispanics (n = 60,683)		
		Non-Hispanic White	Non-Hispanic Black	Mexican/Mexican American	Puerto Rican	Cuban/Cuban American
**Total, n (%)^b^**	427,975 (100%)	298,803 (69.8%)	68,489 (16.0%)	48,093 (79.2%)	8,171 (13.5%)	4,419 (7.3%)
**Sex**						
Male	48.6 (48.4–48.8)	48.7 (48.4–48.9)	54.8 (54.2–55.3)	53.2 (52.6–53.8)	47.8 (46.3–49.4)	50.8 (49.2–52.4)
Female	51.4 (51.2–51.6)	51.3 (51.1–51.6)	42.8 (0.13)	46.8 (46.2–47.4)	52.2 (50.6–53.7)	49.2 (47.6–50.8)
**Age, mean (SE), y^c^**	46.0 (0.07)	47.3 (0.08)	28.6 (0.04)	38.4 (0.14)	42.1 (0.26)	49.0 (0.48)
**BMI, mean (SE), kg/m^2^**	27.2 (0.02)	26.7 (0.02)	28.6 (0.04)	28.0 (0.04)	28.1 (0.09)	27.0 (0.11)
**Education**						
< High school diploma	16.0 (15.7–16.3)	11.7 (11.4–11.9)	20.7 (20.1–21.4)	47.3 (46.4–48.2)	30.4 (28.7–32.1)	26.5 (24.7–28.3)
High school diploma or GED	29.5 (29.2–29.7)	29.7 (29.3–30.0)	31.0 (30.5–31.5)	25.4 (24.8–26.0)	29.2 (27.9–30.5)	25.7 (24.1–27.4)
> High school diploma	54.5 (54.1–55.0)	58.7 (58.2–59.2)	48.3 (47.4–49.2)	27.3 (26.4–28.1)	40.4 (38.6–42.3)	47.8 (45.9–49.7)
**Annual income, $**						
<35,000	58.7 (58.3–59.1)	55.4 (54.9–55.8)	67.7 (66.8–68.6)	76.9 (76.0–77.7)	64.8 (62.8–66.8)	61.8 (59.0–64.6)
35,000–55,000	21.9 (21.6–22.1)	22.8 (22.5–23.0)	20.5 (19.8–21.1)	15.2 (14.5–15.9)	20.9 (19.2–22.7)	20.1 (17.9–22.4)
>55,000	19.4 (19.1–19.7)	21.8 (21.4–22.2)	11.8 (11.2–12.4)	7.9 (7.4–8.4)	14.3 (12.6–15.9)	18.0 (15.4–20.6)
**Health insurance**						
Yes	84.5 (84.3–84.7)	88.3 (88.1–88.5)	79.4 (78.9–79.9)	56.8 (56.0–57.7)	80.8 (79.6–82.1)	77.7 (76.0–79.4)
No	15.5 (15.3–15.7)	11.7 (11.5–11.9)	20.6 (20.1–21.1)	43.2 (42.3–44.0)	19.2 (17.9–20.4)	22.3 (20.6–24.0)

Abbreviations: BMI, body mass index; CI, confidence interval; GED, general equivalency diploma; NHIS, National Health Interview Survey.

a Weighted. All values are % (95% CI) unless otherwise stated.

b Unweighted N.

c *P* < .001.

Annual individual income varied significantly across race/ethnicity (*P* < .001) and Hispanic subgroup (*P* < .001). Overall, more Hispanics (74.5%) than non-Hispanic blacks (67.7%) and non-Hispanic whites (55.4%) had an annual income of less than $35,000. More Mexicans/Mexican Americans (76.9%) had an annual income of less than $35,000 than non-Hispanic blacks (67.7%), Puerto Ricans (64.8%), Cubans/Cuban Americans (61.8%), or non-Hispanic whites (55.4%). Multiple comparison tests showed no significant differences between non-Hispanic blacks and Puerto Ricans at any level of income. Health insurance (any health insurance) was also significantly lower for Mexicans/Mexican Americans (56.8%) than for any other racial/ethnic group. As with income, differences between non-Hispanic blacks, Puerto Ricans, and Cubans/Cuban Americans were not significant.

Similarly, education was significantly associated with race/ethnicity (*P* < .001) and Hispanic subgroup (*P* < .001). Overall, more Hispanics had less than a high school diploma (43.8%) than non-Hispanic blacks (20.7%) and non-Hispanic whites (11.7%). When disaggregated, more Mexicans/Mexican Americans had less than a high school education (47.3%) than Puerto Ricans (30.4%), Cubans/Cuban Americans (26.5%), non-Hispanic blacks, and non-Hispanic whites. Multiple comparison tests were significant for all racial/ethnic comparisons except those between non-Hispanic blacks and Puerto Ricans who reported having a high school diploma or GED and non-Hispanic blacks and Cubans/Cuban Americans who reported having more than a high school diploma (results not shown).

### Heterogeneity by education

We calculated the average annual diabetes prevalence with confidence intervals for all racial/ethnic groups by level of education and *P* values for racial/ethnic differences in average annual diabetes prevalence by level of education (Table 2). Within each level of education, we found a significant difference in the average annual diabetes prevalence (*P* < .001) by race/ethnicity (non-Hispanic white, non-Hispanic black, and Hispanic). Across Hispanic subgroups, differences in average annual diabetes prevalence within each level of education were significant except for the category “greater than a high school diploma.” Among those with less than a high school education, the average annual diabetes prevalence was highest for Puerto Ricans (17.6%). This rate was significantly higher than the average annual diabetes prevalence for Cubans/Cuban Americans (13.4%), non-Hispanic whites (12.1%), and Mexicans/Mexican Americans (9.7%) but did not differ significantly from the prevalence for non-Hispanic blacks (16.1%).

**Table 2:** Average Annual Diabetes Prevalence by Race/Ethnicity and Education, Study of Heterogeneity in Self-Reported Diabetes Prevalence Trends Across 3 Hispanic Subgroups, National Health Interview Survey 1997–2012

Characteristic	< High School Diploma		High School Diploma/GED		> High School Diploma	
	% (95% CI)	*P* Value^a^	% (95% CI)	*P* Value^a^	% (95% CI)	*P* Value^a^
**Race/ethnicity**						
Non-Hispanic white^b^	12.1 (11.7–12.5)	<.001	8.1 (7.9–8.3)	<.001	5.3 (5.2–5.4)	<.001
Non-Hispanic black^b^	16.1 (15.3–16.9)		9.7 (9.2–10.2)		8.2 (7.8–8.6)	
Hispanic^b^^,^ c	10.6 (10.0–11.1)		7.0 (6.6–7.4)		6.6 (6.2–7.1)	
**Hispanic subgroup**						
Mexican/Mexican American^b^	9.7 (9.2–10.3)	<.001	6.3 (5.8–6.9)	<.001	6.7 (6.1–7.2)	.68
Puerto Rican^b^	17.6 (15.7–19.6)		9.8 (8.3–11.2)		6.8 (5.8–7.8)	
Cuban/Cuban American^b^	13.4 (11.3–15.5)		8.2 (6.6–9.8)		6.0 (4.6–7.5)	

Abbreviations: CI, confidence interval; GED, general equivalency diploma.

a *P* for analysis of variance test of race/ethnicity and Hispanic subgroup differences <.001.

b *P* for analysis of variance test of race/ethnicity <.001 and Hispanic subgroup differences = .03.

c All Hispanic ethnic subgroups combined.

Among participants with a high school diploma or GED, the average annual diabetes prevalences for Puerto Ricans (9.8%) and Cubans/Cuban Americans (8.2%) were significantly lower than the annual diabetes prevalences for Puerto Ricans and Cubans/Cuban Americans with less than a high school education. The average annual diabetes prevalence for Puerto Ricans with a high school diploma or GED was not significantly different from that of non-Hispanic blacks with a high school education (9.7%) but was higher than that of non-Hispanic whites (8.1%). Mexicans/Mexican Americans with a high school diploma or GED had average annual diabetes prevalence lower than that of non-Hispanic whites with a high school diploma or GED. Average annual diabetes prevalence for participants with more than a high school diploma was lower for all racial/ethnic groups, yet was highest among non-Hispanic blacks (8.2%) and lowest among non-Hispanic whites (5.3%).

The intercept for the time trend model of annual diabetes prevalence or “baseline prevalence” (β_0_), 5-year time trend (β_5YR_), and *P* values for the time trend in annual prevalence from 1997 to 2012 for racial/ethnic groups were stratified by level of education (Table 3). Among those with less than a high school diploma, the baseline diabetes prevalence was highest among Puerto Ricans (β_0 _= 14.3%), which was almost twice the baseline diabetes prevalence observed for all Hispanics (7.16%) and higher than that for non-Hispanic blacks (12.9%). Mexicans/Mexican Americans had the lowest baseline diabetes prevalence (6.0%). For participants with less than a high school diploma, the time trend for annual age-diabetes prevalence per 5 years was highest among Cubans/Cuban Americans (β_5YR_ = 4.8, *P*_TIME_ = .002). Annual diabetes prevalence significantly increased over time for non-Hispanic blacks (β_5YR_ = 2.2, *P*_TIME_ < .001) and non-Hispanic whites (β_5YR_ = 2.1, *P*_TIME_ < .001). The increasing trend over time in annual diabetes prevalence was not significant for Puerto Ricans.

**Table 3:** Trends Over Time in Annual Weighted Diabetes Prevalence by Race/Ethnicity and Education, Study of Heterogeneity in Self-Reported Diabetes Prevalence Trends Across 3 Hispanic Subgroups, National Health Interview Survey, 1997–2012

Race/Ethnicity	< High School Diploma			High School Diploma/GED			> High School Diploma		
	β_0_	β_5YR_	*P *Value	β_0_	β_5YR_	*P *Value	β_0_	β_5YR_	*P *Value
Non-Hispanic white	9.1	2.1	<.001	5.0	2.1	<.001	3.2	1.3	<.001
Non-Hispanic black	12.9	2.2	<.001	6.7	2.0	<.001	5.5	1.7	<.001
Hispanic^a^	7.5	1.9	<.001	5.1	1.1	.001	4.1	1.5	<.001
Mexican/Mexican American	6.0	2.2	<.001	4.4	1.1	.005	4.4	1.3	.001
Puerto Rican	14.3	2.2	.061	6.8	1.8	.032	2.4	2.6	.001
Cuban/Cuban American	6.6	4.8	.002	5.4	1.8	.173	5.6	0.4	.610

Abbreviations: β_0_, time trend model intercept; β_YR_, 5-year trend in diabetes prevalence; GED, general equivalency diploma.

a All Hispanic ethnic groups combined.

Among participants with a high school diploma or GED, baseline diabetes prevalence (β_0_) for Puerto Ricans and Cubans/Cuban Americans was lower than the baseline prevalence for Puerto Ricans and Cubans/Cuban Americans with less than a high school education. The time trend for diabetes prevalence per 5 years was highest among non-Hispanic whites (β_5YR_ = 2.1, *P*_TIME_ < .001) and Puerto Ricans (β_5YR_ = 1.8, *P*_TIME_ = .032). The 5-year trend was lowest among Mexicans/Mexican Americans (β_5YR_ = 1.1, *P*_TIME_ = .005). Baseline diabetes prevalence among participants with more than a high school diploma was lower for all racial/ethnic groups than for participants with less than a high school education or a high school diploma/GED. However, despite having the lowest baseline diabetes prevalence, Puerto Ricans had the highest time trend for annual diabetes prevalence per 5 years (β_5YR_ = 2.6, *P*_TIME_ = .001).

## Discussion

Our study is the first to disaggregate US Hispanic subgroups to demonstrate disparities in national diabetes prevalence trends over time. The disparities in diabetes prevalence for Hispanic subgroups compared with non-Hispanic whites and non-Hispanic blacks were initially masked in our study by the use of a pan-ethnic group that included all Hispanics. In these national data, the disparities vary significantly by Hispanic subgroup. As a pan-ethnic group, Hispanics have an annual diabetes prevalence lower than that of non-Hispanic blacks and slightly higher than that of non-Hispanic whites. When prevalence by Hispanic subgroup is considered, Puerto Ricans and non-Hispanic blacks have similar annual diabetes prevalence with sizeable increases over the 15-year time frame. The disparity between Mexicans/Mexican Americans and non-Hispanic whites was less pronounced than the disparities seen for Puerto Ricans and non-Hispanic blacks compared with non-Hispanic whites.

We observed even more variations in diabetes trends by race/ethnicity and education. Although the trend over time for Puerto Ricans was higher than those for all other racial/ethnic groups, among those with more than a high school diploma, diabetes prevalence was lowest for Puerto Ricans than for all other racial/ethnic groups with more than a high school diploma. This finding suggests that over the 15-year time frame of this study, Puerto Ricans with more than a high school diploma saw a marked increase in the prevalence of diabetes. However, at both the high school diploma/GED and more than a high school diploma levels, diabetes prevalence for non-Hispanic blacks was higher than that of Puerto Ricans.

Previous studies that examined Hispanic subgroup differences in diabetes prevalence found similar overall diabetes prevalence rates for Puerto Ricans, Mexicans/Mexican Americans, and Cubans/Cuban Americans (19–21). However, in those studies, Mexicans and Mexican Americans were considered 2 separate subgroups and 2 additional subgroups of Dominican and Central/South American were used for analysis, as opposed to the 3 largest Hispanic subgroups we used in our study (19–21). Pabon-Nau et al (21) restricted their study population to Hispanic adults, using Mexican Americans as the referent group, to examine the effect of Hispanic subgroup on the odds of diabetes by using pooled NHIS data from 2000 to 2005. Borrell et al (19) included non-Hispanic whites and non-Hispanic blacks in their study, as did we, and used non-Hispanic whites as the reference group in their analyses to assess the strength of association between race/ethnicity and self-reported diabetes using pooled NHIS data from 1997 to 2005. Despite the differences in composition of the study population, the aggregated estimates we obtained were similar to previously published estimates. However, those studies did not look at disparities over time.

Given the scope of our study, we did not focus our analysis on estimating the association between race/ethnicity and diabetes, which has been explored in previous research (19–21). Instead, we focused on describing and testing racial/ethnic disparities in diabetes prevalence over time. At the time of analysis, only 1 other study had focused on health disparities and differences across Hispanic subgroups over time; however, the outcome of the study was cancer mortality, not diabetes, and the study population was restricted to people living in Florida (17). Our findings regarding diabetes prevalence support the implications of Martinez-Tyson et al (17) that presenting health outcomes (ie, cancer death rates) for Hispanics as a pan-ethnic group often masks the differences between the ethnic subgroups falling under that pan-ethnic Hispanic umbrella.

Several limitations in this study warrant consideration. First, diabetes prevalence is estimated by using self-reported diabetes. However, self-reported diabetes and other health outcomes have excellent agreement with medical records (26–29). Also, the NHIS self-reported diabetes question also does not differentiate between type 1 and type 2 diabetes. Despite the difference, CDC and the National Institutes of Health use these data to produce national estimates of type 2 diabetes prevalence (1) because type 2 accounts for 90% to 95% of diabetes diagnoses. Given the large sample size in our study, a potential misclassification of participants with type 1 diabetes as having type 2 diabetes is unlikely to affect the trends seen in the self-reported diabetes prevalence over time. We excluded data on participants who reported borderline diabetes from the final analysis to reduce the potential for misclassification bias. We conducted a post hoc sensitivity analysis in which we kept these participants categorized as borderline as “no” for self-reported diabetes. The analysis revealed that our results are robust with respect to the definition of the outcome (self-reported diabetes) and exclusion of participants with borderline diabetes. Most surveys, including national surveys, are conducted in English with on-the-spot and irregular translation into Spanish, resulting in Hispanic respondents with limited English proficiency being excluded in past national surveys. Because ad hoc translations could severely limit the representativeness of survey data, the NHIS instrument was translated into Spanish in 1998 and has been available in Spanish since then (30). Given that the data in the present study were taken from 1997 to 2012, this particular limitation does not pose a serious threat to the representativeness of Hispanics in these data. Also, although we acknowledge the importance of including the 2 additional specific Hispanic subgroups (Dominican and Central/South American) and the “other Hispanic” subgroup, we did not include these subgroups because of concerns over the annual sample sizes available for these groups.

Our results support the need to disaggregate surveillance data for Hispanics in the United States and have implications for projections that may be used to allocate resources for targeting specific subgroups in health promotion initiatives and to improve monitoring and surveillance to equitably quantify reductions in the burden of diabetes over time. Given the low education level of some groups, counseling and materials to support prevention and management of diabetes should be tailored for low literacy. Racial/ethnic variations in the trends over time for other related health outcomes and modifiable risk factors such as obesity, hypertension, and physical activity also require further research. Although the specific mechanisms by which education affects diabetes prevalence at the national level need further exploration, our results suggest that evidence-based, culturally appropriate targeted interventions to improve high school graduation rates, college enrollment, and retention can benefit all racial/ethnic groups.
